# Trifocal Femoral Fracture Treated With an Intramedullary Nail Accompanied With Compression Bolts and Lag Screws: Case Presentation and Literature Review

**DOI:** 10.7759/cureus.8173

**Published:** 2020-05-17

**Authors:** Ioannis Papaioannou, Andreas Baikousis, Panagiotis Korovessis

**Affiliations:** 1 Orthopedics, General Hospital of Patras, Patras, GRC; 2 Orthopedics and Traumatology, General Hospital of Patras, Patras, GRC; 3 Orthopedics and Traumatology and Spine Surgery, General Hospital of Patras "Agios Andreas", Patras, GRC

**Keywords:** combined femoral fractures, type 4, nail, modification, compression bolts

## Abstract

Ipsilateral combined fractures of the proximal femur, femoral shaft, and distal femur occur rarely with few published cases in the literature. These injures are classified as type 4 combined femoral fractures according to the classification of Lambiris et al. We present a rare case of a combined injury including an ipsilateral intertrochanteric fracture, a mid-shaft transverse femoral fracture, and a Y-shaped intra-articular fracture of distal femur in a 36-year-old man following a traffic accident. There was also an un-displaced extra-articular fracture of the ipsilateral patella. This combined injury has been reported only once, while our treatment strategy has never been reported in the literature. We used a single long Gamma-nail to treat all three fractures, while we locked the nail distally with compression bolts. The intra-articular part of the distal femoral fracture was managed with two cannulated percutaneous 6.5 mm lag screws. This modification of the nail allowed us to lock the nail and also to compress the metaphyseal part of the distal femoral fracture and secure this fracture to the nail. Our patient had an uneventful recovery, while the union was observed to all fractures four months postoperatively. As these combined femoral injuries are rare, there is no consensus of the management of such fractures. Many authors suggest an individualized approach to these rare cases based on the configuration of all fractures, especially the proximal and the distal one. By this case presentation we cite an alternative treatment of type 4 combined femoral fractures. Trauma surgeons may benefit from this Gamma-nail modification for such complicated injuries.

## Introduction

Ipsilateral combined fractures of the hip, femoral shaft, and distal femur occur very rarely with only few reported cases [1]. Even rarer is the combination of a mid-shaft femoral fracture combined with an extra-articular proximal femur fracture and a Y-shaped intra-articular distal femoral fracture accompanied with a fracture of the ipsilateral patella that was first reported in 2004 [2]. To the best of our knowledge, this is the second case with this combination of fractures, but the first case treated with a single long Gamma-nail accompanied with additional cancellous lag screws and compression bolts. In this article we describe our surgical strategy and technique, while a review of the existing literature is also cited.

## Case presentation

**Figure 1:**
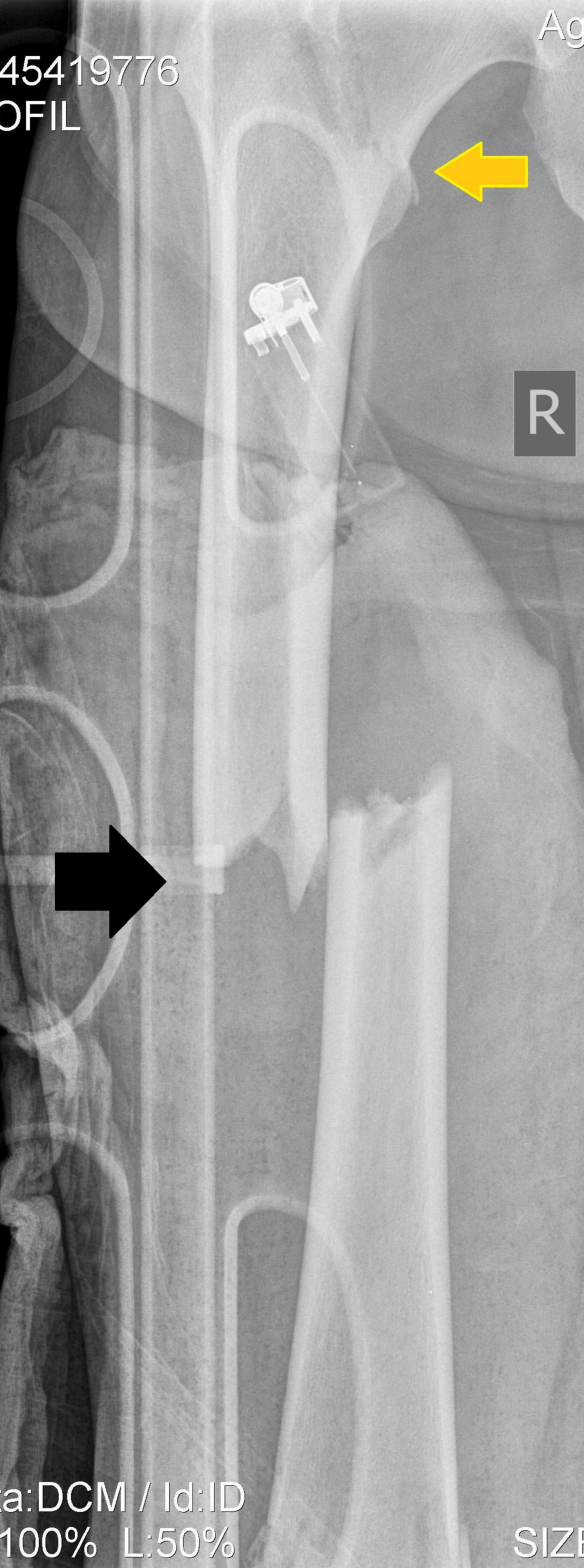
Anteroposterior radiogram of the right femur demonstrates the mid-shaft femoral fracture (black arrow), while the ipsilateral proximal femoral fracture can be easily seen (yellow arrow).

**Figure 2:**
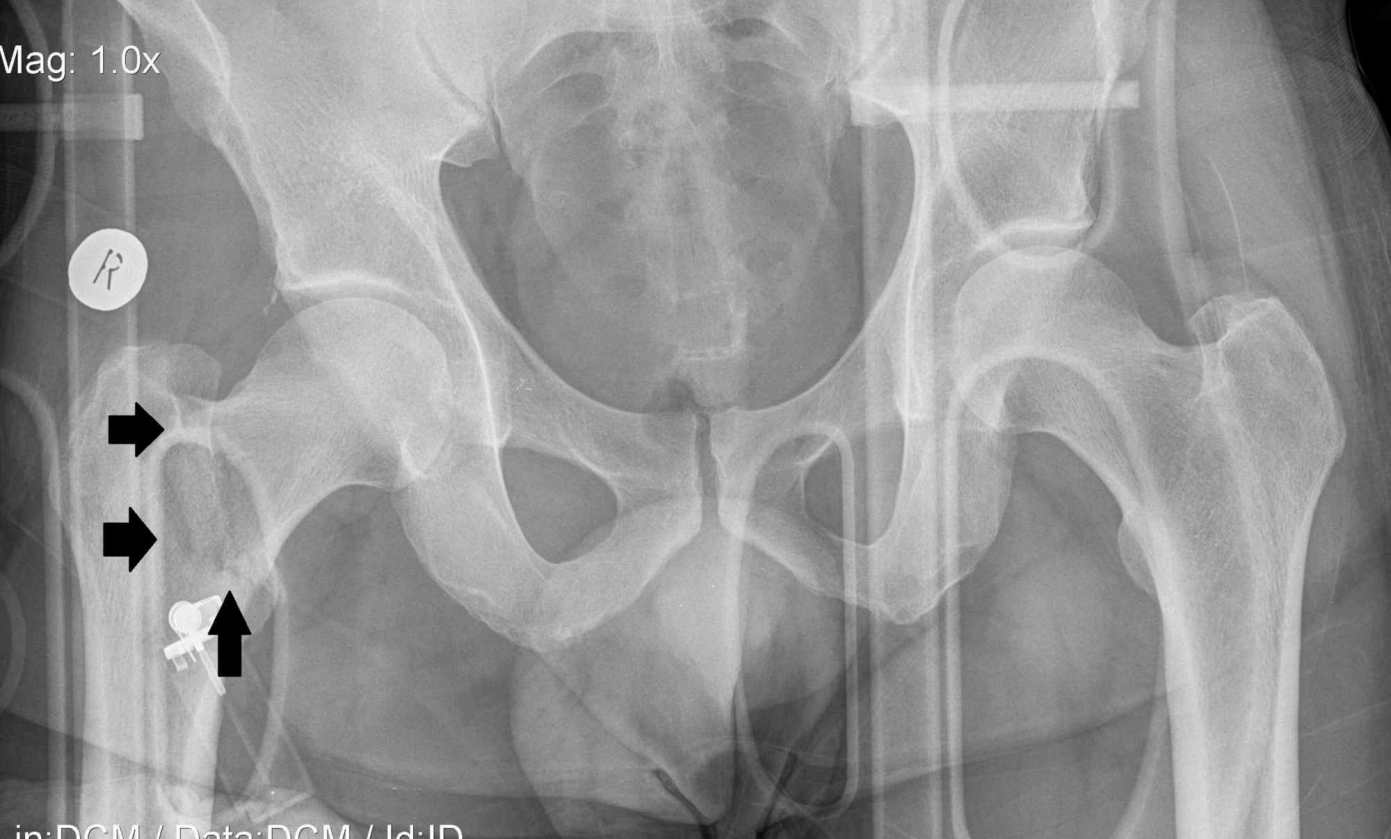
Anteroposterior radiogram of right hip demonstrates the proximal intertrochanteric fracture (black arrows).

**Figure 3:**
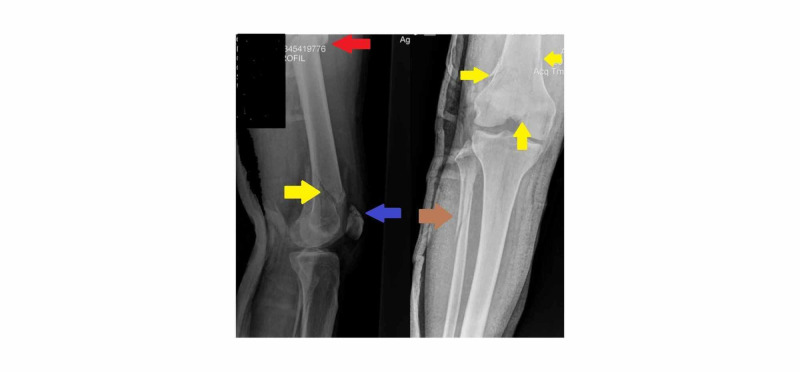
Profile and anteroposterior radiogram of distal femur and knee demonstrate the intra articular distal femoral fracture (yellow arrows), the fracture of the patella (blue arrow), the fibula (brown arrow), and the femoral shaft (red arrow).

A 36-year-old man was admitted to our ED after a high energy motor vehicle accident. Physical examination and X-rays revealed a displaced transverse right femoral mid-shaft fracture 32 Α3/ AO type (Figure 1), a nondisplaced intertrochanteric 31 A1/AO type fracture (Figure 2) as well as an intra-articular fracture of the distal femur of AO type 33 C1/AO. The distal femur injury was a high Y-shaped intra-articular fracture, with minimal articular surface displacement and slight displacement to the metaphyseal region. Furthermore, an extra-articular incomplete fracture of the proximal pole of ipsilateral patella 34 A1/AO type and a fracture of the proximal third of ipsilateral fibula shaft of AO type 4F2.B/AO were also revealed based on radiographic examination (Figure 3).

 

 

**Figure 4:**
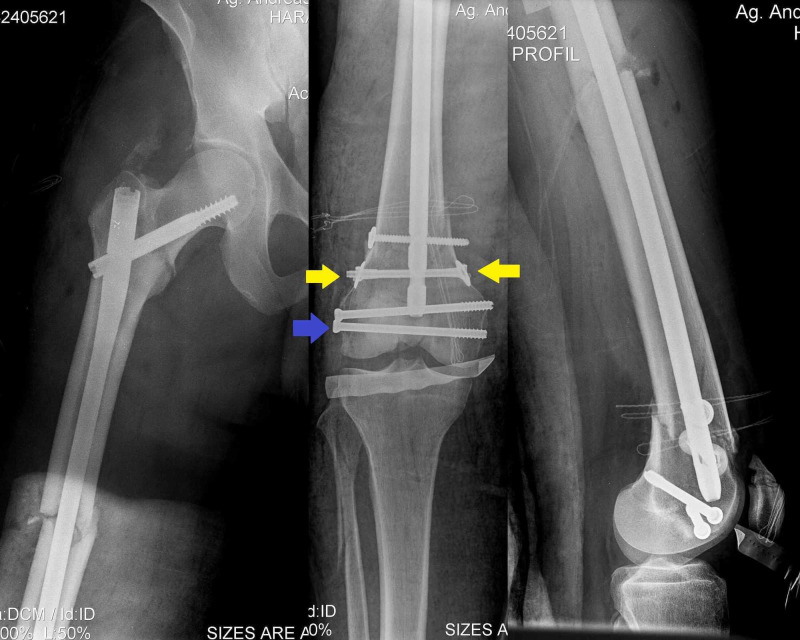
Anteroposterior and profile postoperative X-rays demonstrate the long Gamma-nail accompanied with compression bolts (yellow arrows) and cannulated lag screws (blue arrow).

All injuries were closed, the patient was hemodynamically stable, and he was operated 24 hours after admission. Our concern was the intra-articular distal femur fracture, so before placing the patient to the traction table we preferred to fix this fracture with cannulated 6.5 mm partially treated cancellous screws to compress and stabilize this fracture under biplane image intensifier. Afterwards, we applied axial traction from the tibial tubercle and we decided to manage all the ipsilateral fractures with a Gamma-3 Long Nail (Stryker, Mahwah, NJ, USA) using also the compression bolts. The compression bolts (Stryker, Mahwah, NJ, USA) were used to lock the nail and also to compress the metaphyseal Y-shaped fracture of the distal femur fracture (Figure 4).

**Figure 5:**
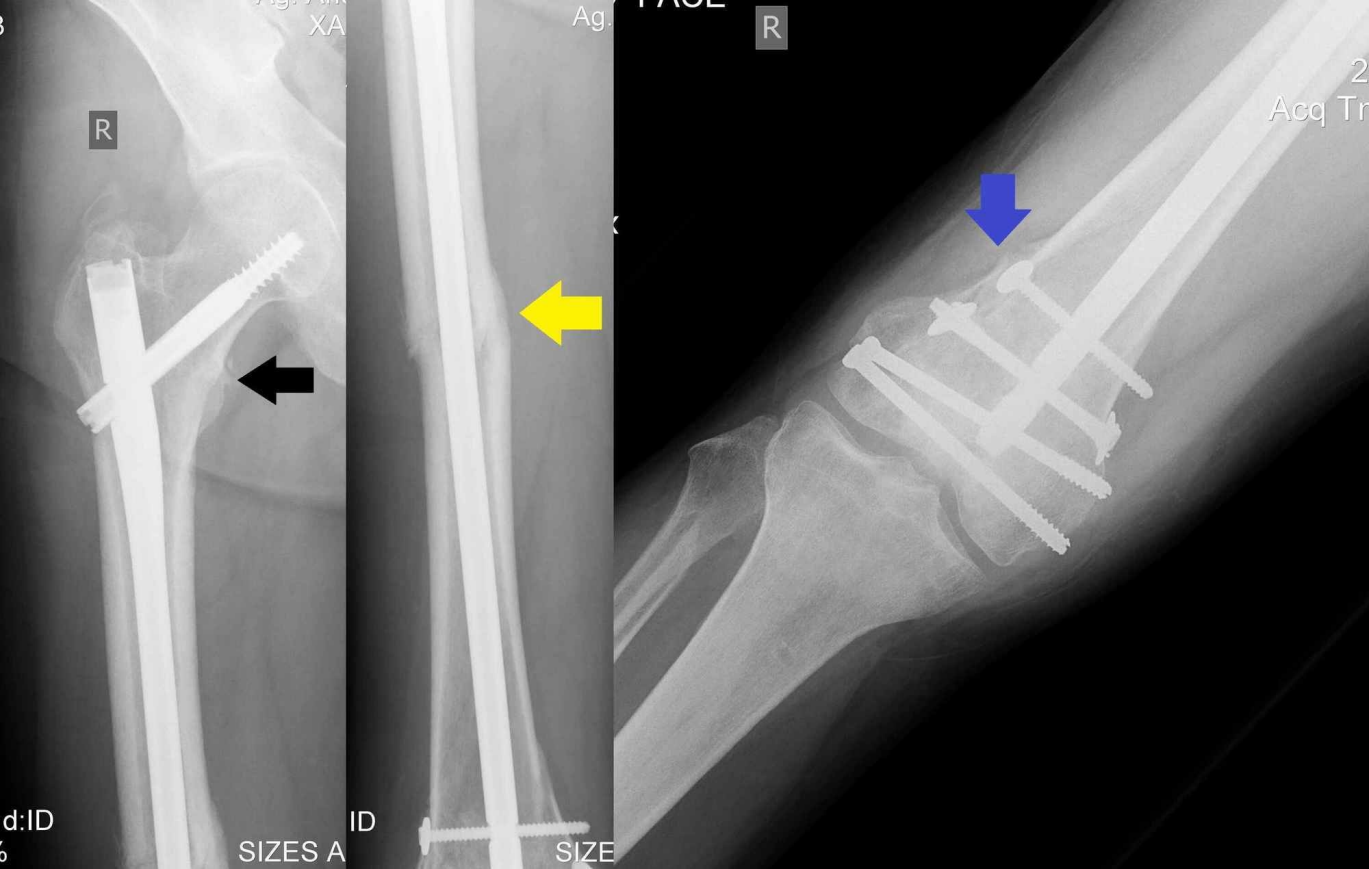
Postoperative anteroposterior X-ray of right femur at four months follow up demonstrates union of all three fractures (proximal femur-black arrow, femoral shaft-yellow arrow, and distal femur-blue arrow).

**Figure 6:**
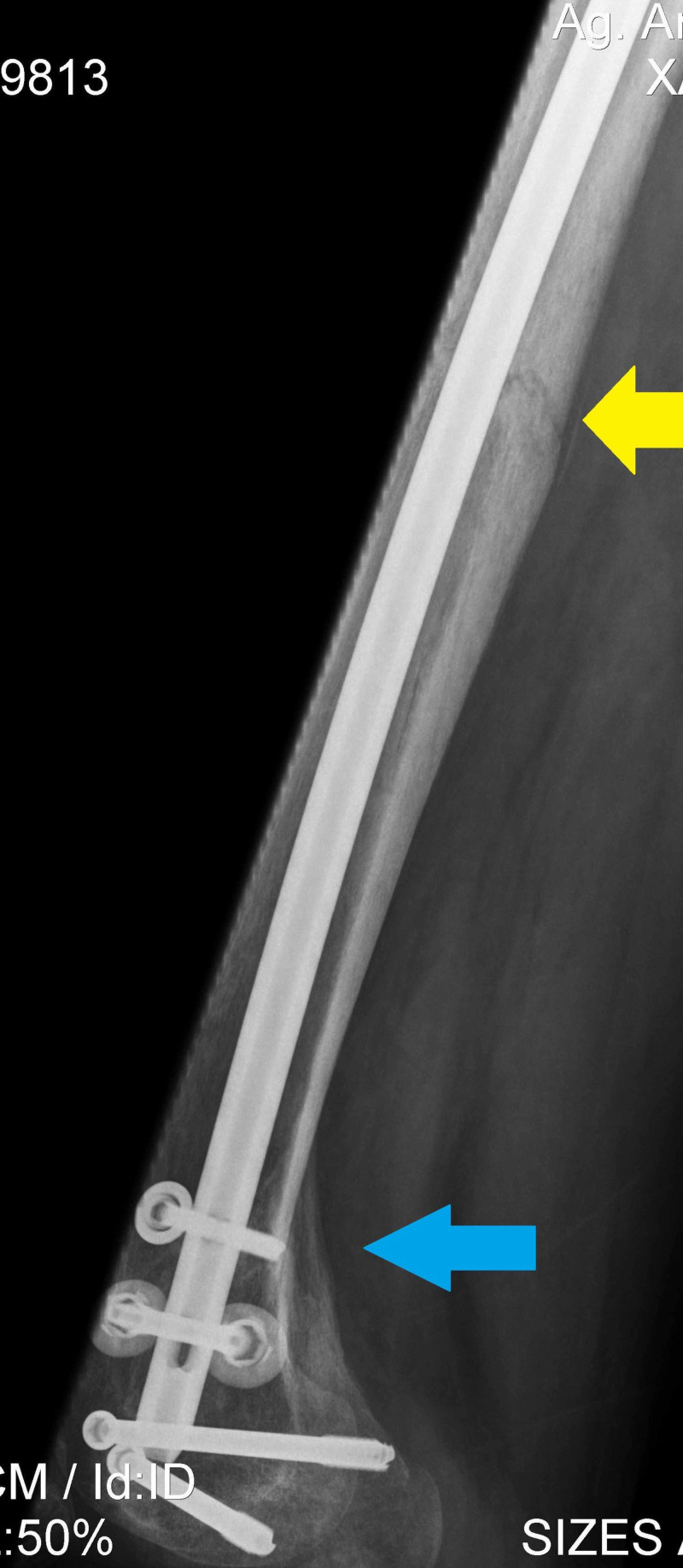
Postoperative profile X-ray of right femur at four months follow up demonstrates union of shaft (yellow arrow) and distal femur fracture (blue arrow).

**Figure 7:**
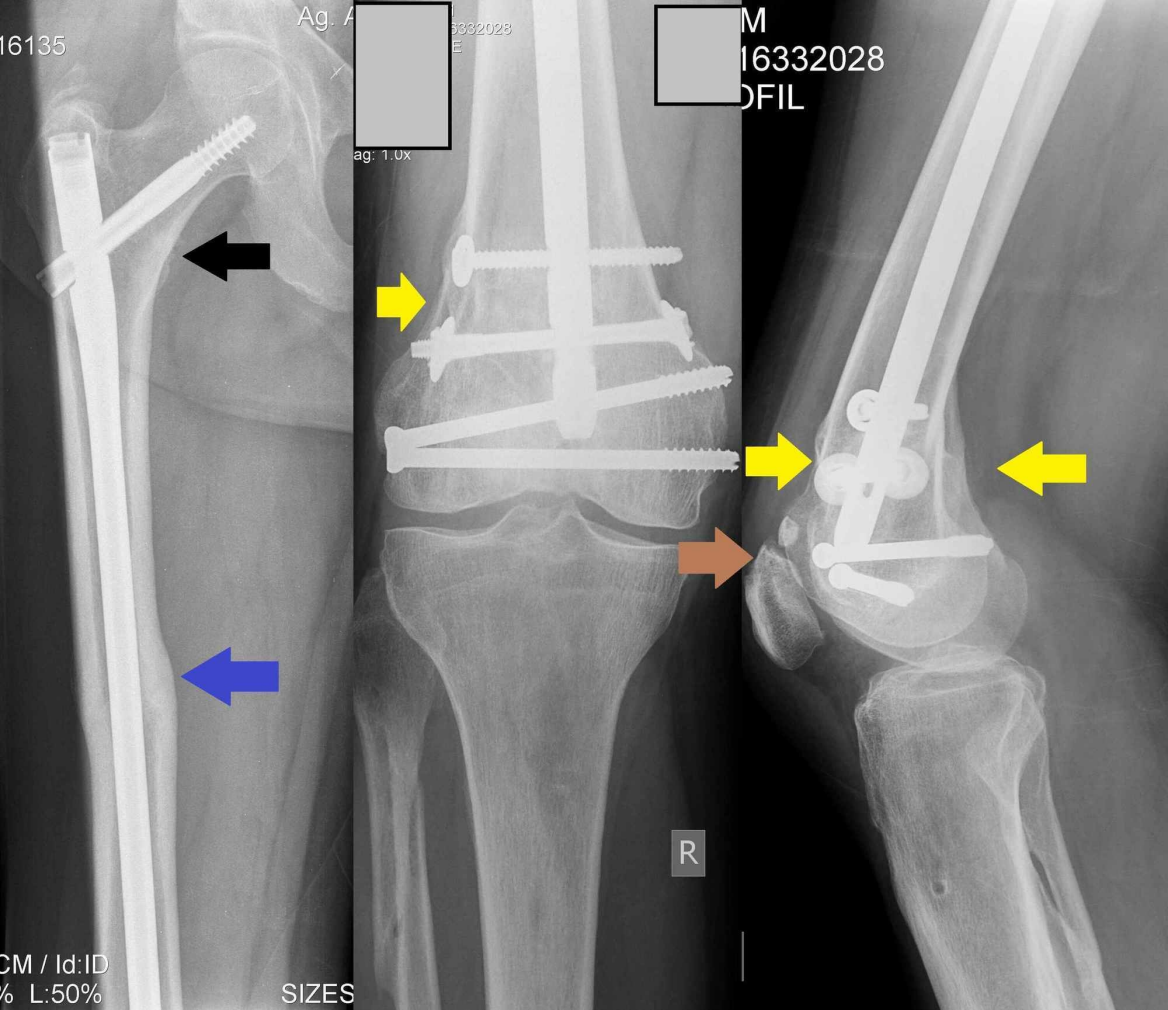
Postoperative X-rays of right femur at one year follow up demonstrate union and remodeling of all fractures (proximal femur-black arrow, femoral shaft-blue arrow, distal femur-yellow arrows, and patella-brown arrow).

By this modification of the nail accompanied with the cannulated screws we intended to fix all the fractures with a single device, respecting the soft tissues, based on the minimal invasive techniques of intramedullary nail implantation. Our surgical technique was based on the preoperative planning and the successful stabilization of the distal fracture via the lag screws was contributed to the implementation of our plan. After fixation of the femoral fractures, we evaluated the stability of the patella fracture and no displacement was observed even with 100o of flexion. The knee joint ligaments were found stable. Our patient had an uneventful recovery and was discharged six days after the initial admission. A knee brace was placed to our patient, the knee mobilization began after three weeks, and partial weight-bearing started one month postoperatively. The follow up at four months postoperatively revealed union on all fractures (Figures 5-6) accompanied with excellent function of hip and knee joint, while the Oxford Knee Score (46/48) and the Harris Hip Score (100/100) were excellent. The patient returned to his routine six months after surgery and the follow up at 14 months revealed excellent union and remodeling of all fractures (Figure 7).

 

.

## Discussion

The combination of hip fracture, femoral shaft, and distal femoral fracture occurs very rarely [3-4]. In 2003 a classification was proposed for these combined femoral fractures [5]. Type 4 combined femoral fractures are the rarest combination and to best of our knowledge, until now only 21 cases with type 4 combined femoral fractures have been described in the literature [1-9]. These cases have been treated with many different fixation techniques, while there is no report to our fixation strategy. Regarding the proximal femoral fractures, we found nine cases with intra-capsular fractures, five cases with extra-capsular fractures, five cases with per-trochanteric fractures, and two cases with sub-trochanteric fractures. The majority of the diaphyseal fractures were located in the mid-shaft. Concerning the distal femoral fractures, the vast majority were type B (11 cases), while the fracture of the lateral femoral condyle was dominant among them. Finally, we found four cases with supracondylar fracture and six cases with intra-articular distal femoral fracture. Three of these 21 cases had also an ipsilateral patellar fracture, as in our case [1-2, 7]. To the best of our knowledge, our case is the second presented to the literature with this combination of fractures; intertrochanteric fracture, mid-shaft femoral fracture, intra-articular distal femoral fracture, and fracture of the patella [2]. Type 4 combined femoral fractures are very challenging injuries, because the combination of techniques and utilization of multiple implants is frequently required to achieve optimal fixation for all three fractures. Many authors suggest an individualized approach to these rare cases based on the configuration of all fractures, especially the proximal and the distal one. Regarding the proximal femoral fracture, dynamic hip screw (DHS) [1, 6, 9], cannulated screws [3, 6, 7], and reconstruction intramedullary nails [2] have been used from the authors. The femoral shaft fracture was treated with a nail in the vast majority, except six cases [1, 6, 9], in which authors faced this fracture with plating treating also the distal femoral component. The distal femoral fracture was treated with lag screws [1, 4, 6-8], 95o blade plate [1-3, 6], reconstruction nail [5, 8], and anatomical locking plate [9]. Finally, the patella was treated with cerclage in two cases [1] and with a lag screw in one case [7]. After reviewing the literature, we noticed that in several cases multiple implants have been used leaving “free bone”, causing stress concertation to this area. If possible, fixation of all fractures with a single device accompanied using minimal invasive technique is desired. To the best of our knowledge, the technique that was described in this case report has never been reported in the literature. Although, our technique is not applicable for all type 4 combined fractures, the main limitations are the displacement of the distal femoral fracture and the metaphyseal extension of this fracture. In case of a displaced or a very low transverse distal femoral fracture, open reduction and internal fixation with long plate to address also the femoral shaft fracture is required and subsequently the proximal femoral fracture should be addressed via lag screws or DHS device. This alternative has two major disadvantages; the "open" technique and the "free bone" between the two plates. It is worth noting, that in our technique the lag screws should be placed as low as possible to avoid the interference with the nail. So, in our case we faced all the fractures with a long Gamma-3 nail accompanied with two lag screws. The difference between our technique and the published data concerning the fixation strategy of these challenging fractures was the usage of compression bolts (cannulated 5 mm screw with nuts to both sides) and this modification allowed us to lock the nail, to compress the metaphyseal part of the distal femoral fracture, and to secure this fracture to the nail.

## Conclusions

Combined femoral fractures are rare injuries, while great awareness and adequate radiographic evaluation are mandatory for diagnosis. Trauma surgeons should be aware of this modification and its application. We believe that the modified surgical technique with Gamma-nail and lag screws can achieve excellent outcomes to these severe combined femoral fractures due to the minimally invasive technique, which dramatically reduces the possibility of fracture nonunion and subsequently the revision surgery.
